# Preparation of the standard cell lines for reference mutations in cancer gene-panels by genome editing in HEK 293 T/17 cells

**DOI:** 10.1186/s41021-020-0147-2

**Published:** 2020-02-11

**Authors:** Takayoshi Suzuki, Yoshinori Tsukumo, Chie Furihata, Mikihiko Naito, Arihiro Kohara

**Affiliations:** 1 Division of Molecular Target and Gene Therapy Products, National Institute of Health Sciences, 3-25-26, Tonomachi-ku, Kawasaki, 210-9501 Japan; 2 JCRB Cell Bank, National Institutes of Biomedical Innovation, Health and Nutrition, 7-6-8, Saito-Asagi, Ibaraki City, Osaka, 567-0085 Japan

**Keywords:** Next generation sequencer, Genome editing, CRISPR/Cas9, Mutant standard, Cancer gene panel

## Abstract

**Background:**

Next Generation Sequencer (NGS) is a powerful tool for a high-throughput sequencing of human genome. It is important to ensure reliability and sensitivity of the sequence data for a clinical use of the NGS. Various cancer-related gene panels such as Oncomine™ or NCC OncoPanel have been developed and used for clinical studies. Because these panels contain multiple genes, it is difficult to ensure the performance of mutation detection for every gene. In addition, various platforms of NGS are developed and their cross-platform validation has become necessity. In order to create mutant standards in a defined background, we have used CRISPR/Cas9 genome-editing system in HEK 293 T/17 cells.

**Results:**

Cancer-related genes that are frequently used in NGS-based cancer panels were selected as the target genes. Target mutations were selected based on their frequency reported in database, and clinical significance and on the applicability of CRISPR/Cas9 by considering distance from PAM site, and off-targets. We have successfully generated 88 hetero- and homozygous mutant cell lines at the targeted sites of 36 genes representing a total of 125 mutations.

**Conclusions:**

These knock-in HEK293T/17 cells can be used as the reference mutant standards with a steady and continuous supply for NGS-based cancer panel tests from the JCRB cell bank. In addition, these cell lines can provide a tool for the functional analysis of targeted mutations in cancer-related genes in the isogenic background.

## Introduction

Innovative advances in DNA sequencing technology have deepened the understanding of cancer genetic abnormalities and accumulated huge volumes of data on genetic abnormalities in various human cancers [1–4]. On the other hand, the so-called molecular targeted drugs, targeting specific cancer-related genes, have been developed based on the genetic alterations observed in human cancers [5]. Diagnosis of a genetic abnormality is becoming indispensable for deciding whether to administer a drug, which is known as the companion diagnosis [6–8] for the molecular targeted drugs in which genetic abnormalities and therapeutic action of the drugs are clearly linked. Last few years have witnessed increasing the clinical application of the so-called “cancer gene panels” [9, 10] for a comprehensive analysis of multiple genetic abnormalities. At the same time application of the next-generation sequencers (NGS), which is expected as a useful tool for realizing genomic medicine on human cancer, is gaining popularity. For its clinical application, however, it is necessary to ensure the reliability and sensitivity of the sequence data for multiple genes [11, 12]. But it is difficult to guarantee the performance of mutation detection for all installed genes in the comprehensive cancer gene panel. Such cancer panel tests consist of multiple steps, including sample preparation, nucleic acid extraction, library preparation for sequence analysis, and hardware (NGS) and software for sequence determination, and making it difficult to ensure validity for all steps. Therefore a standard method to validate the process of the whole diagnosis system by using the standard material is advocated [13].

Synthetic DNAs of known sequence can be used as a standard for validation of a DNA sequencer itself [14], but it is desirable to use a pathological specimen closer to real clinical samples in order to validate the whole processes of diagnosis including sample preparation [11]. When the number of gene to be sequenced is limited, a portion of the clinical specimen can be preserved and used as the reference material for the specific gene, but it is difficult to prepare such standards for all the genes in the panel. Further, for several genetic diseases, authentic clinical samples are not available due to rarity of the mutation. Therefore, development of similar standard products is desired to serve as useful tools in the development and validation of test systems. In addition, from the viewpoint of steady supply, purity (heterogeneity), and coverage of mutations, it is difficult to utilize FFPE specimen in a long run. Therefore, the established cell line, which is considered to be closer to clinical samples than synthetic DNA has been proposed to maintain a steady supply of the homogeneous and more reliable reference material covering whole range of mutations in the cancer gene panels. If the cultured cells can be used, it is also possible to combine variety of gene mutations, which is particularly desirable as a standard for the cancer gene panels.

In order to create such mutant standards for versatile cancer-related genes, we have used a genome-editing technology with CRISPR/Cas9 [15, 16], which is recently getting popular, and tried to integrate known mutations of interest into a defined cell line. The human embryonic kidney derived cell line, HEK 293 T/17 cell, which is frequently used for genome-editing because of a high efficiency, was used. Details of pathogenic and high frequency mutations reported in the COSMIC database [17] was retrieved, guide RNAs were designed for those mutations and appropriate knock-in strains were created by genome editing. In this process, since the clinical application of NCC oncopanel [18], developed by the National Cancer Research Center, was progressed, we decided to select those genes that are included in the COSMIC database but missing in the existing cell lines in JCRB. Construction of a cell line mixture covering all the 114 genes in the NCC OncoPanel will be reported in a separate manuscript.

In this article, we describe introduce the creation of the genome edited strains and their properties, and discuss about their usage including a use for the comprehensive standard for the NCC OncoPanel.

## Materials and methods

### Cells

The human embryonic kidney HEK 293 T/17 cells were obtained from ATCC Manassas, VA, USA). The cells were cultured in DMEM (Sigma-Aldrich) supplemented with 10% FBS (Thermo Fisher Scientific)) and 1% penicillin-streptomycin (Thermo Fisher Scientific). Absence of mycoplasma was checked by the MycoAlert Mycoplasma Detection Kit (Lonza) occasionally.

### Selection of the target mutations in cancer-related genes

Cancer-related genes that are frequently used in the cancer gene panels, or those found in the Japanese mutation database (REF [19]) were selected as the candidate genes and details on mutations reported for those genes were searched in the COSMIC database [1]. In the candidate genes, those mutations which are reported to be pathogenic and found with higher frequency were selected and also considering adjacent Cas9-target sequence of PAM (protospacer adjacent motif) site (3′ NGG) for Cas9 cleavage. Then the guide RNA (gRNA), which contains a complemental sequence of the targeting site, was designed for the mutation. The possibility of off-target effect was checked by the GGGnome [2] software and those with higher off-targets were avoided.

### Genome-editing by CRISPR/Cas9

For the genome-editing, the DNA-directed RNA-guided endonuclease (RGEN) system (TakaraBio) was used [20]. Designed gRNA sequence was integrated into the expression vector under the U6 promotor (pRGEN_U6_SG). Cas9 endonuclease was integrated into the expression vector under the CMV promotor (pRGEN-Cas9-CMV). These plasmids were transfected into *E.coli* and purified by the NucleoBond Xtra Midi EF (Macherey-Nagel). The 71–78 bp long single-stranded oligonucleotide (ssODN) (100 pmol) was transfected along pRGEN_U6_SG (0.17 μg) and pRGEN-Cas9-CMV (0.25 μg) vectors into 1.75 × 10^5^ HEK 293 T/17 cells by using TransIT-X2 reagent (Mirus Bio). Then the cells were cultured in 24 well tissue culture plates for 3 days. As an alternative method for 4 genes (AKT3, BIM, IGF2 and MYCN), gRNA was prepared by in vitro transcription (IVT) using Guide-it sgRNA In Vitro Transcription Kit (Takara) and was transfected with Cas9 protein by the Neon Transfection System (Thermo Fisher Scientific) with two pulses of 1100 V and 20 ms.

Regarding the method of introducing the Cas9, a transfection of the Cas9 proteins as a complex with gRNA (RNP) was used in the later experiment [20], instead of the standard expression vector method.

### Performance of gRNA assessed by T7E1 assay

After 3 days, a portion of the culture cells was subjected to the T7E1 assay [21]. Genomic DNA was prepared from the cells by the Nucleospin Tissue Kit (Macherey-Nagel) and the targeted region was PCR amplified by Tks Gflex DNA Polymerase (TakaraBio) with the appropriate primers (94 °C for 1 min; (98°Cfor 10s, 60 °C for 15 s, 68 °C for 30s) for 30 cycles; kept at 4 °C) in the TP600 thermal cycler (TakaraBio). The PCR products (20 μl) were purified using the NucleoSpin Gel and PCR Clean-up kit (Macherey-Nagel), heat denatured and re-annealed by the TP600 thermal cycler (94 °C for 2 min; 85 °C for 1 s; 30 °C for 10 min; kept at 4 °C). Then 2 μl of T7 Endonuclease 1 (10 U/μl; New England Biolabs) was added and incubated at 37 °C for 30 min. The reaction was stopped by adding 1 μl of 0.5 M EDTA. The digested DNA fragments were analyzed by the Agilent 1000 kit in 2100 Bioanalyzer (Agilent Technologies). When the digested fragment was detected, the cells were subjected further to the single cell cloning.

### Cloning and screening of the targeted mutations

The cells were harvested by 0.25% Trypsin-EDTA (Thermo Fisher Scientific) to make a single cell suspension and plated into 96 Half Area Well Clear Flat Bottom TC-Treated Microplate (Sigma-Aldrich) at a density of 0.4 cells/well. Then cells were expanded by transferring into 24 well tissue culture plates and each clone was stored in CELLBANKER1 at − 80 °C until DNA sequence analysis and subsequent stoke.

For the sequence analysis, the cell pellet was subjected to the Single Prep reagent for DNA (TakaraBio) and the prepared genomic DNA was used for the amplification of targeted regions by the corresponding primers. After purification of the PCR products by Sephadex G-50 Fine column (GE Healthcare), DNA sequence was analyzed by the BigDye Terminator v3.1 Cycle Sequencing Kit (Thermo Fisher Scientific) with the forward primer for amplification.

## Results

The selected target genes and mutation sites are summarized in Table 1. Although the panel of 39 target genes was selected mainly from NCC OncoPanel [22], those with more overlap with other similar cancer gene panels (Oncomine [23], Illumina Trusight tumor 26 [24], Personalis [25], and Ion Ampliseq Cancer Hotspot panel [26]) were also included. Target site for mutation was basically selected from the COSMIC mutations registered with higher frequency and pathogenicity and with a consideration of the Japanese database (NBDC) [19]. Then the gRNA was designed for the target site closest to a PAM site and with less chance of off-target. Number of 1–3 mismatch sites in genome was screened by the GGGnome software. Single strand oligo DNA (ssODN) adjacent to the target site was prepared with a length of 71–78 bp. Based on the gRNA sequence, expression vector pRGEN_U6_SG was constructed for each target gene which was co-transfected with the Cas9 expression vector pRGEN-Cas9-CMV and ssODN.

**Table 1:** List of targeted genes, site of mutations, and design of gRNA

Gene	NCC Oncopanel (ver 2)	Thermo Fisher Oncomine Dx Test	Personails ACE CancerPlus™ Test	Illumina Trusight Tumor 26	Ion AmpliSeq Cancer Hotspot Panel v2	No of mutations in COSMIC	COSMIC_ID	CDS Mutation	FATHMM		gRNA sequense	(No of mismatch)				
												Distanse from PAM	0	1	2	3
AKT1	*	*	*	*	*	975	COSM33765	49G > A	Pathogenic	(score 1.00)	CACCACCCGCACGTCTGTAGGGG	1	1	1	1	12
AKT3	*		*			269	COSM242892	232C > A	Pathogenic (score 0.99)		TCTCTATAACAGTAGTCCACTGG	1	1	0	0	27
ALK	*	*	*	*	*	1522	COSM28056	3824G > A	Pathogenic	(score 0.98)	TACTCACCTGTAGATGTCTCGGG	4	1	1	1	16
BAP1	*		*			1221	COSM110721	178C > T	Pathogenic	(score 0.98)	CCAAGGTAGAGACCTTTCGCCGG	5	1	1	1	9
BCL2L11(BIM)	*		*			116	COSM389356	585G > C	Pathogenic (score 0.98)		GTTACATTGTCCGCCTGGTGTGG	1	1	0	0	7
BRAF	*	*	*>	*	*	27,630	COSM476	1799 T > A	Pathogenic (score 0.99)		TAGCTACAGTGAAATCTCGATGG	12	1	0	1	20
BRAF’	*	*	*	*	*	4	COSM1137	1817G > A	Pathogenic (score 0.98)		ACAGTGAAATCTCGATGGAGTGG	1	1	1	0	25
CDK4	*	*	*			101	COSM1677139	70C > T	Pathogenic	(score 0.98)	AGTGGCCACTGTGGGGATCACGG	2	1	1	2	45
CDKN2A(p16)	*	*	*		*	5911	COSM12475	238C > T	Pathogenic	(score 0.88)	GGGCAGCGTCGTGCACGGGTCGG	2	1	1	1	4
CTNNB1(β-catenin)	*	*	*	*	*	7307	COSM5664	121A > G	Pathogenic	(score 0.98)	CAGAGAAGGAGCTGTGGTAGTGG	6	1	1	11	195
DNMT3A		*	*			3679	COSM52944	2645G > A	Pathogenic	(score 0.98)	CGTCTCCAACATGAGCCGCTTGG	6	1	1	2	18
ERBB2(HER2)	*	*	*	*	*	1596	COSM48358	929C > T	Pathogenic	(score 0.97)	CAGGGGGCAGACGAGGGTGCAGG	1	1	1	21	201
ERBB3	*	*	*			900	COSM20710	310G > A	Pathogenic	(score 0.88)	ACCATTGCCCAACCTCCGCGTGG	4	1	1	1	7
EZH2	*	*	*		*	1273	COSM37028	1937A > T	Pathogenic	(score 0.99)	GAATTCATCTCAGAATACTGTGG	7	1	1	5	77
FBXW7	*	*		*	*	1972	COSM22975	1513C > T	Pathogenic	(score 0.94)	TGCCATCATATTGAACACAGCGG	2	1	1	1	18
FGFR3	*	*	*		*	4354	COSM715	746C > G	Pathogenic	(score 0.96)	CTGCAGGATGGGCCGGTGCGGGG	1	2	4	7	58
FOXL2		*		*		933	COSM33661	402C > G	Pathogenic	(score 0.95)	CTTCTCGAACATGTCTTCGCAGG	5	1	1	1	12
HRAS	*	*	*		*	2023	COSM502	183G > T	none	(score 0.53)	CATCCTGGATACCGCCGGCCAGG	2	2	2	2	16
IDH2	*	*	*		*	2430	COSM33733	515G > A	Pathogenic	(score 0.99)	CCAAGCCCATCACCATTGGCAGG	2	1	1	4	86
IGF2	*					132	COSM1561457	293C > T	Pathogenic (score 0.97)		ACCCTCACCGGAAGCACGGTCGG	2	1	0	0	1
JAK2	*	*	*		*	50,556	COSM12600	1849G > T	Pathogenic	(score 0.94)	AATTATGGAGTATGTGTCTGTGG	8	1	1	1	75
KIT	*	*	*	*	*	8856	COSM1314	2447A > T	Pathogenic	(score 0.99)	AGAATCATTCTTGATGTCTCTGG	7	1	1	1	133
KNSTRN		*				118	COSM140056	71C > T	Neutral	(score 0.00)	GTAGCTAGGCGGAAGTGGGTGGG	1	1	1	2	28
KRAS	*	*	*	*	*	43,548	No ID	124G > A	–		CTTGTGGTAGTTGGAGCTGGTGG	4	1	1	1	46
MAGOH		*				43	COSM535605	410 T > C	Pathogenic	(score 0.99)	TGTTTGGTCTTCAGTCTTATTGG	4	1	2	3	76
MAP 2 K1	*	*	*	*		496	COSM235614	370C > T	Pathogenic	(score 0.99)	CCATAGAAGCCCACGATGTACGG	1	1	4	4	10
MAPK1		*	*			173	COSM461148	964G > A	Pathogenic	(score 0.98)	GTATTACGACCCGAGTGACGAGG	4	1	1	1	2
MDM2	*		*			110	COSM431747	994C > T	Pathogenic	(score 0.91)	AGGAAGCCAATTCTCACGAAGGG	6	1	1	2	25
MET	*	*	*	*	*	1142	COSM707	3029C > T	Pathogenic	(score 0.98)	TTTGAAACCATTTCTGTAGTTGG	5	1	1	2	78
MTOR	*	*	*			1195	COSM20417	6644C > A	Pathogenic	(score 1.00)	CCAATGACCCAACATCTCTTCGG	8	1	1	1	12
MYCN	*					305	COSM35624	131C > T	Pathogenic (score 0.96)		CTTCCAGATGTCCTCCCCCGGGG	1	1	0	0	24
NOTCH1	*	*	*		*	3921	COSM12771	4799 T > C	Pathogenic	(score 0.99)	CCACGTTGGTGTGCAGCACGCGG	9	1	1	1	53
NRAS	*	*	*	*	*	6676	COSM5930625	112G > A	Pathogenic	(score 0.99)	AAACTGGTGGTGGTTGGAGCAGG	1	1	1	1	67
PDGFRA	*	*	*	*	*	2354	COSM736	2525A > T	Pathogenic	(score 0.99)	CGAATCATGCATGATGTCTCTGG	7	1	1	1	24
PIK3CA	*	*	*	*	*	13,613	COSM775	3140A > G	Pathogenic	(score 0.96)	ATGAATGATGCACATCATGGTGG	1	1	0	1	56
PTCH1	*	*	*			1331	COSM1638394	3944C > T	Pathogenic	(score 1.00)	AGCGTCTCTGCGCGGTCTGTAGG	1	1	1	1	7
PTEN	*	*	*	*	*	4778	No ID	697C > T	–		AACTTGTCTTCCCGTCGTGTGGG	7	1	0	1	7
SMO	*	*	*		*	593	COSM216037	1234C > T	Pathogenic	(score 0.96)	AGCCTCCCACGATGAGCACCAGG	8	1	1	1	102
STAT3	*	*				805	COSM1155743	1919A > T	Pathogenic	(score 0.97)	TTCAGCTGCTGCTTTGTGTATGG	5	1	1	6	92
TP53(p53)	*	*	*	*	*	39,196	COSM10662	743G > A	Pathogenic	(score 0.98)	GCATGGGCGGCATGAACCGGAGG	5	1	0	0	3

**Fig. 1:**
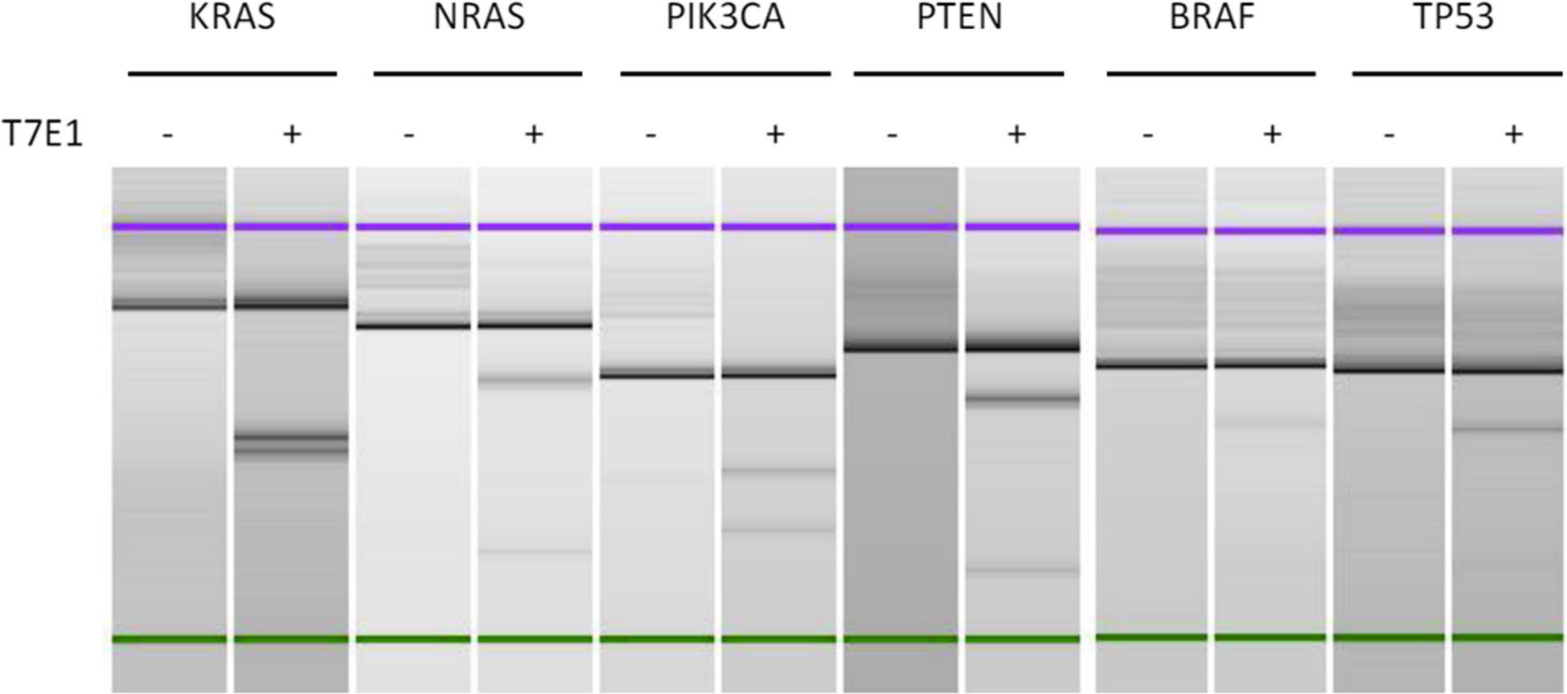
Results of T7E1 assay. After transfection of expression vectors pRGEN_U6_SG, pRGEN-Cas9-CMV and ssODN corresponding to the target genes (KRAS, NRAS, PIK3CA, PTEN, BRAF and TP53), genomic DNA was isolated from the transfected cells. The target region was amplified by PCA with corresponding primers. After denature and re-annealing of the PCR product, it was digested by T7 endonuclease I (T7E1) which cut mismatched DNA fragments. Cleaved band suggests an introduction of mutations by ssODN

Effectiveness of gRNA to cut the targeted DNA sequence was monitored by the T7E1 assay using the genomic DNA isolated from the transfected cells. Figure 1 shows results of T7E1 assay for KRAS, NRAS, PIK3CA, PTEN, BRAF and TP53 genes. When the predicted DNA fragments were obtained in the T7E1 assay, the cells were taken for further cloning. In the case of negative results, the gRNA was re-designed or the target gene was changed.

**Fig. 2:**
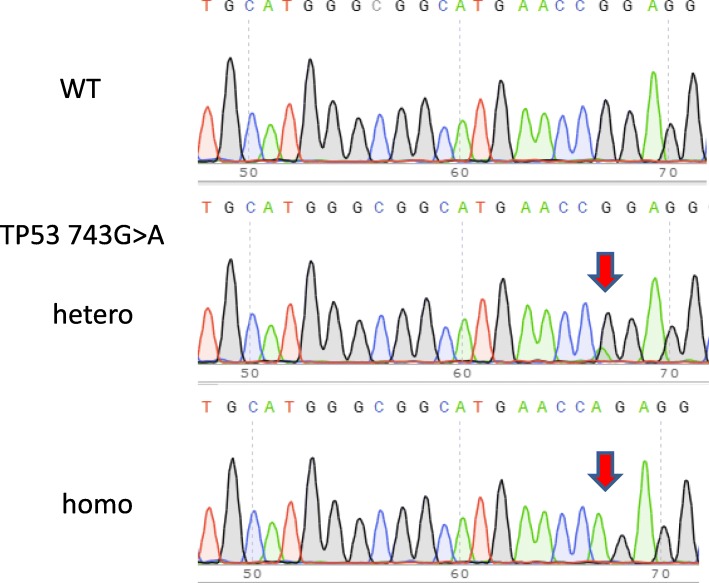
Confirmation of the TP53 743 G > A mutation by Sanger sequencing. Existence of the targeted knock-in mutation was checked for each isolated clones by the Sanger sequencing after PCR amplification of the target region. Successful examples for TP53 743 G > A mutation are shown. Both homo and hetero knock-in mutants were obtained

Finally 36 out of 39 target genes were preceded for colony isolation and sequence determination. Due to the inability of using a marker for knock-in mutant selection, more than 150 clones were screened for each gene by the direct Sanger sequencing of PCR products (Fig. 2). Because HEK 293 T/17 cell is having a near triploid karyotype with various copy number among the whole chromosome region, it was difficult to fully diagnose the sequence spectrum. The targeted event was judged as homozygote or heterozygote as shown in Table 2. The latter includes other than 1:1 ratio depending on the chromosomal location but the exact number of the mutant allele was not determined. Because of the unstable karyotype, the exact copy number should be validated before being used as a reference cell line. It can be thought that the copy number of whole population is stable at least within a few passages during a reference preparation.

**Table 2:** Targetted mutations and recoveries of the knock-in mutants

Method	Gene	Mutation	bp from PAM	Total Clones	Homo Knock-in	Hetero Knock-in	Targeted Rate(%)
Vector	AKT1	49 G > A	1	177	0	0	0.0
Vector	ALK	3824 G > A	4	190	4	5	6.8
Vector	BAP1	178 C > T	5	183	0	12	6.6
Vector	CDKN2A	238 C > T	2	156	5	2	7.7
Vector	CTNNB1	121 A > G	6	164	1	0	1.2
Vector	DNMT3A	2645 G > A	6	149	0	1	0.7
Vector	ERBB3	310 G > A	4	171	0	1	0.6
Vector	EZH2	1937 A > T	7	153	4	0	5.2
Vector	FBXW7	1513 C > T	2	167	0	0	0.0
Vector	FGFR3	746 C > G	1	167	0	1	0.6
Vector	HRAS	183 G > T	2	172	2	5	5.2
Vector	IDH2	515 G > A	2	155	0	7	4.5
Vector	JAK2	1849 G > T	8	164	3	1	4.3
Vector	KIT	2447 A > T	7	172	3	12	10.5
Vector	KNSTRN	71 C > T	1	161	1	0	1.2
Vector	KRAS	35 G > A	4	160	0	2	1.3
Vector	MAGOH	410 T > C	4	160	0	5	3.1
Vector	MAP 2 K1	370 C > T	1	177	1	0	1.1
Vector	MAPK1	964 G > A	4	169	0	0	0.0
Vector	MDM2	994 C > T	6	171	0	2	1.2
Vector	MET	3029 C > T	5	154	1	1	1.9
Vector	MTOR	6644 C > A	8	182	2	9	7.1
Vector	NOTCH1	4799 T > C	9	167	0	2	1.2
Vector	NRAS	35 G > A	1	157	2	0	2.5
Vector	PDGFRA	2525 A > T	7	176	7	6	11.4
Vector	PIK3CA	3140A > G	1	161	0	1	0.6
Vector	PTEN	697 C > T	7	156	0	1	0.6
Vector	SMO	1234 C > T	8	157	8	7	14.6
Vector	STAT3	1919 A > T	5	161	0	1	0.6
Vector	TP53	743 G > A	5	159	2	2	3.8
Protein	BRAF	1817 G > A	1	172	1	16	10.5
Protein	AKT3	232 C > A	2	192	0	5	2.6
Protein	BIM	585 G > C	1	192	6	9	10.9
Protein	IGF2	293 C > T	2	192	0	6	3.1
Protein	MYCN	1132 G > A	1	192	10	26	24.0

Together with the targeted knock-in event, many deletion mutations were observed around the targeted site; it happened simultaneously in different allele in some cases. Off-target mutations near the targeted site were also observed in few cases. An improvement in editing efficiency was seen in RNP experiments (average success rate increased to 10.2 from 3.5, *p* = 0.0055 by T-test) (Table 2).

Initially five clones each for 6 genes (NRAS, KRAS, PIK3CA, PTEN, TP53 and BRAF) and then two clones each for the rest were selected and stored after expansion of the culture. Out of 36 genes taken for trial, the targeted mutation (either homo or hetero) could be obtained for 33 genes with more than 90% success rate. The mutant clones having more variations in each gene were selected. Finally, a panel of 88 isolated clones of HEK 293 T/17 cells representing 125 mutations in total was produced including some non-targeted mutants (Table 3).

**Table 3:** List of Mutant Cell Lines Created by Genome Editting in HEK293T/17 Cell

Target Gene	Cell Clone Name (ID)	JCRB Cell ID	Mutation detail^a^
AKT1	49 G>A	293T-AKT1-1	delTG(43-44) Homo
		293T-AKT1-2	49G>A(COSM33765)hetero+55insT&49G>A(COSM33765)Hetero
AKT3	232C>A	293T-AKT3-1	232C>A(COSM242892)Hetero+del231-238(8bp) Hetero+236delG&235T>A Hetero
		293T-AKT3-2	232C>A(COSM242892)Hetero
ALK	3824 G>A	293T-ALK-1	3824G>A(COSM28056)Homo
		293T-ALK-2	3824G>A(COSM28056)Hetero
BAP1	178 C>T	293T-BAP1-1	178C>T(COSM110721)Hetero
		293T-BAP1-2	178C>T(COSM110721)Hetero&160-173(14bp) duplicate
BIM	585G>C	293T-BIM-1	585G>C(COSM389356)Homo+590 insTHetero
		293T-BIM-2	585G>C(COSM389356)Hetero
BRAF	1817G>A	293T-BRAF-1	1817G>A(COSM1137)Hetero
		293T-BRAF-2	1817G>A(COSM1137)Hetero
		293T-BRAF-3	1817G>A(COSM1137)Hetero+1820delC&1817G>A(COSM1137)Hetero
		293T-BRAF-4	1817G>A(COSM1137)Hetero+1811insG Hetero
		293T-BRAF-5	1817G>A(COSM1137)Homo
CDKN2A	238 C>T	293T-CDKN2A-1	238C>T(COSM12475)Hetero
		293T-CDKN2A-2	238C>T(COSM12475)Homo
CTNNB1	121 A>G	293T-CTNNB1-1	121A>G(COSM5664)Homo+del116-9&112G>A Hetero+del114-9 Hetero
		293T-CTNNB1-2	121A>G(COSM5664)Homo
DNMT3A	2645 G>A	293T-DNMT3A-1	2643insC Homo
		293T-DNMT3A-2	2645G>A(COSM52944)Hetero
ERBB3	310 G>A	293T-ERBB3-1	306insC Homo
		293T-ERBB3-2	310G>A(COSM20710)Hetero
EZH2	1937 A>T	293T-EZH2-1	1937A>T(COSM37028)Homo+1831T>C Homo
		293T-EZH2-2	1937A>T(COSM37028)Homo
FBXW7	1513 C>T	293T-FBXW7-1	1518insT Homo
		293T-FBXW7-2	1518insT Hetero+1518insCA Hetero
FGFR3	746 C>G	293T-FGFR3-1	752insA Homo
		293T-FGFR3-2	746C>G(COSM715)Hetero
HRAS	183 G>T	293T-HRAS-1	183G>T(COSM502)Homo
		293T-HRAS-2	183G>T(COSM502)Hetero
IDH2	515 G>A	293T-IDH2-1	515G>A(COSM33733)Hetero+512G>A(COSM86960) Hetero
		293T-IDH2-2	515G>A(COSM33733)Hetero
IGF2	293C>T	293T-IGF2-1	293C>T(COSM1561457)Hetero&del276-289(14bp)Hetero +294-295insCA+del270-283(14bp)Hetero
		293T-IGF2-2	293C>T(COSM1561457)Hetero
JAK2	1849 G>T	293T-JAK2-1	1849G>T(COSM12600)Homo
		293T-JAK2-2	1849G>T(COSM12600)Hetero
KIT	2447 A>T	293T-KIT-1	2447A>T(COSM1314)Homo
		293T-KIT-2	2447A>T(COSM1314)Hetero
KNSTRN	71 C>T	293T-KNSTRN-1	71C>T(COSM140056) Homo
		293T-KNSTRN-2	71C>T(COSM140056) &Del67-69(3bp) Homo
KRAS	35 G>A	293T-KRAS-1	32insC Homo+35G>A(COSM521)Hetero+del30-38(9bp) Hetero
		293T-KRAS-2	35G>A(COSM521)Hetero+33delT Hetero
		293T-KRAS-3	35G>A(COSM521)Hetero
		293T-KRAS-4	35G>A(COSM521)&30-33AGCT>CGTA Hetero+32insC Hetero
		293T-KRAS-5	35G>A(COSM521)&del20-34(15bp)Hetero+32insCC Hetero+32insC Hetero
MAGOH	410 T>C	293T-MAGOH-1	410T>C(COSM535605)Hetero
		293T-MAGOH-2	410T>C(COSM535605)&407delT Hetero
MAP2K1	370 C>T	293T-MAP2K1-1	370C>T(COSM235614)Hetero+370C>T(COSM235614)Hetero&376insA hetero+358-377(20bp) duplicate Hetero
		293T-MAP2K1-2	376insA Homo
MAPK1	964 G>A	293T-MAPK1-1	960insG Homo
		293T-MAPK1-2	del962-968(7bp) Hetero+del962-969&4bp(12bp) Hetero+del926-969&69bp(113bp) Hetero
MDM2	994 C>T	293T-MDM2-1	995insG Homo
		293T-MDM2-2	994C>T(COSM431747)Hetero
MET	3029 C>T	293T-MET-1	3029C>T(COSM707)Hetero
		293T-MET-2	3029C>T(COSM707)Homo
MTOR	6644 C>A	293T-MTOR-1	6644C>A(COSM20417)Hetero
		293T-MTOR-2	6644C>A(COSM20417)Homo
MYCN	1132 G>A	293T-MYCN-1	1132G>A(COSM229914)Homo
		293T-MYCN-2	1132G>A(COSM229914)Hetero+1126delG Hetero
NOTCH1	4799 T>C	293T-NOTCH1-1	4799T>C(COSM12771)Hetero
		293T-NOTCH1-2	4797insG Homo
NRAS	35 G>A	293T-NRAS-1	35G>A(COSM564)Homo
		293T-NRAS-2	35G>A(COSM564)Homo
		293T-NRAS-3	34G>T(COSM562)Hetero
		293T-NRAS-4	35G>A(COSM564)Hetero+30-31AG>GAAA Hetero
		293T-NRAS-5	35G>A(COSM564)Hetero+del30-44(15bp) Hetero
PDGFRA	2525 A>T	293T-PDGFRA-1	2525A>T(COSM736)Homo
		293T-PDGFRA-2	2525A>T(COSM736)Hetero
PIK3CA	3140A>G	293T-PIK3CA-1	3140A>G(COSM775)Hetero+3143insA Hetero
		293T-PIK3CA-2	3144T>G(COSM27157)Homo
		293T-PIK3CA-3	3140A>G(COSM775)Hetero+del3131-42(12bp) Hetero
		293T-PIK3CA-4	3140A>G(COSM775)Hetero
		293T-PIK3CA-5	3140A>G(COSM775)Hetero+del3102-47(46bp) Hetero
PTEN	697 C>T	293T-PTEN-1	63-64 CG>(T,C,G)(T,C,G)(A,G) (ins 1bp for 3 alleles)
		293T-PTEN-2	697C>T(COSM5154)Hetero
		293T-PTEN-3	697C>T(COSM5154)Hetero+697insA Hetero+697-698CG>TT Hetero
		293T-PTEN-4	697insGorA Hetero
		293T-PTEN-5	697C>T(COSM5154)Hetero&Del 546-672(127bp)&546 5bp ins(535-539) & Del697-715(19bp) Hetero +WT
SMO	1234C>T	293T-SMO-1	1234C>T(COSM216037)Homo
		293T-SMO-2	1234C>T(COSM216037)Hetero
STAT3	1919 A>T	293T-STAT3-1	1921insA Homo
		293T-STAT3-2	1919A>T(COSM1155743)Hetero
TP53	743 G>A	293T-TP53-1	743G>A(COSM10662)Homo+746G>A(COSM44091)Homo
		293T-TP53-2	743G>A(COSM10662)Hetero
		293T-TP53-3	743G>A(COSM10662)Homo
		293T-TP53-4	743G>A(COSM10662)Homo
		293T-TP53-5	743G>A(COSM10662)&746delG Homo

^a^& means mutations were found in the same strand, + for other cases

## Discussion

With the progress made with the genome editing technology using CRISPR/Cas9, it has become possible to modify the genes of interest at relatively easy manner. We applied this technique to prepare a panel of cell lines in which a known gene mutation has been introduced into a target site to use as a standard reference material for genetic diagnosis. Since genome editing was carried out for 39 genes, the basic data obtained in this study could be analyzed for improving genome editing efficiency. The designing of gRNA can be discussed as an important factor in genome editing.

The PAM site, which is a cleavage site by Cas9 protein, is known to be important to initiate genome editing, and it is desirable to select the mutation of interest close to the PAM site [27]. In the standard method of using a Cas9 expression vector, it was predicted that it is desirable to select the site of the target mutation closer to the PAM site. But our data demonstrated that even when it is designed in the vicinity of a PAM site, the genome editing efficiency is not necessarily high, and we could get the mutants even when it is far from the PAM site (up to 9 bp). Therefore, genome editing efficiency was not affected so much by the distance from the PAM site (Correlation coefficient between bp from PAM site and targeted rate is 0.025). However, it is necessary to stay within a certain distance from the PAM site, and it is important to check in advance whether the target site is cleaved by Cas9 and gRNA, using the T7E1 assay, for example. Because we did not use any selective marker gene, we proceeded to the next step only when the cleavage was confirmed. We discontinued the target or changed the design of gRNA for those genes that did not yield clear cleaved bands in the T7E1 assay. In the case of the BRAF gene, although changing target site was effective, but we had to compromise with a relatively low mutation frequency. In the case of prioritizing the site of mutation, it is necessary to increase the efficiency of genome editing. When the “knock-in” cells are necessary, markers such as drug resistance genes can’t be used although that strategy is effective for a simple knockout. In such case, we propose to use the Piggy-Bac system [28], which was once utilized as the replacement for the drug resistance genes for the selection of target, and excises them with transposase to obtain the desired knock-in mutants. By using this method, we have successfully generated the RB1 mutation knock-in strain in suspension cells (Human lymphoblastoid TK6) which is generally difficult for transfection. (unpublished data).

Regarding the design of gRNA, those with few homologous sites on the genome should be selected in order to prevent nonspecific cleavage as much as possible. We have used gRNA design with one or two base mismatches where ever possible, but considerable number of three base mismatches could not be avoided [29, 30]. It is not clear how such sequence similarity affected the off-target event because we only confirmed the sequence of the target sites. It may be necessary to analyze the presence of such an off-target mutation when we characterize the phenotype of the genome-edited cells, but it is not necessary for the purpose of this study to prepare the standard cell lines (DNA) for the particular mutation. For a few genes, constant mutations other than targeted event were observed which suggest SNP in original HEK 293 T/17 strain. It should be noted that this cell line has a p53 mutation derived from large-T antigen treatment as reported [31].

The average genome-editing efficiency for knock-in mutations was around 4.5% which enabled knock-in mutant detection from 150 clones for sequencing. Regarding CRISPR/Cas9 method, Cas9 protein transfer method was also used for the later study in addition to the standard method with the Cas 9 expression vector. The Cas9 protein transfer method improved the genome-editing efficiency.

It is also important to know the exact amount of the mutated alleles and their alterations during culture. It is necessary to quantitatively analyze the dosage of mutations of the standard products using the RT-PCR, the digital PCR, etc. in future.

Although the main purpose of this study is to prepare the standard reference cell lines, the created cell lines can also be used for a functional analysis of mutated genes. Since it was made with the same background of the HEK 293 T/17 cell, it is possible to compare the biological effects of introduced gene mutations with each other including hetero and homo status in some cases. Furthermore, by introducing additional gene mutations in the current strains, it can be utilized to analyze the interaction between two genes and their involvement in the process of carcinogenesis. When the NGS-based cancer gene panel tests are widely used and novel mutations with little clinical information are found, we may face a problem to distinguish whether the mutation is relevant for carcinogenesis or susceptible to certain drugs. We hope the cell-based assay such as a test for proliferation or tumorigenesis using the genome-edited cells will be developed and that will contribute additional data for the decision of clinical procedure.

Finally, the genome-edited cell lines prepared in this study can be used as a mutant standard for each target gene, which is supplied from the JCRB cell bank. These cells will also be supplied as a mixture in the future, as an all-in-one standard for cancer gene panel tests. We also hope they will be used as a standard for a cross validation between different cancer gene panels, NGS platforms, facilities or examiners.

## Conclusions

In this study, we created a panel of genome edited cells for the genes frequently mutated and used in cancer gene panels such as NCC OncoPanel. These cell lines are useful for analytical validation of NGS based cancer gene panel assay. They will also be useful for a cross-platform validation of the different panels, instrument platforms, and examiners as a common standard.

## Data Availability

All the representative clones isolated by genome-editing in HEK 293 T/17 cells have deposited and available at the JCRB cell bank.

## References

[1] Meyerson M, Gabriel S, Getz G (2010). Advances in understanding cancer genomes through second-generation sequencing. Nat Rev Genet.

[2] Wakai T, Prasoon P, Hirose Y, Shimada Y, Ichikawa H, Nagahashi M (2019). Next-generation sequencing-based clinical sequencing: toward precision medicine in solid tumors. Int J Clin Oncol.

[3] Schwartzberg L, Kim ES, Liu D, Schrag D (2017). Precision oncology: who, how, what, when, and when not?. Am Soc Clin Oncol Educ Book.

[4] Sabour L, Sabour M, Ghorbian S (2017). Clinical applications of next-generation sequencing in Cancer diagnosis. Pathol Oncol Res.

[5] Kumar B, Singh S, Skvortsova I, Kumar V (2017). Promising targets in anti-cancer drug development: recent updates. Curr Med Chem.

[6] Milne CP, Bryan C, Garafalo S, McKiernan M (2015). Complementary versus companion diagnostics: apples and oranges?. Biomark Med.

[7] Jorgensen JT, Hersom M (2016). Companion diagnostics-a tool to improve pharmacotherapy. Ann Transl Med.

[8] Jorgensen JT (2016). Companion and complementary diagnostics: clinical and regulatory perspectives. Trends Cancer.

[9] Nagahashi M, Shimada Y, Ichikawa H, Kameyama H, Takabe K, Okuda S (2019). Next generation sequencing-based gene panel tests for the management of solid tumors. Cancer Sci.

[10] Luthra R, Patel KP, Routbort MJ, Broaddus RR, Yau J, Simien C (2017). A targeted high-throughput next-generation sequencing panel for clinical screening of mutations, gene amplifications, and fusions in solid tumors. J Mol Diagn.

[11] Jennings LJ, Arcila ME, Corless C, Kamel-Reid S, Lubin IM, Pfeifer J (2017). Guidelines for validation of next-generation sequencing-based oncology panels: a joint consensus recommendation of the Association for Molecular Pathology and College of American pathologists. J Mol Diagn.

[12] Ma X, Shao Y, Tian L, Flasch DA, Mulder HL, Edmonson MN (2019). Analysis of error profiles in deep next-generation sequencing data. Genome Biol.

[13] Hardwick SA, Deveson IW, Mercer TR (2017). Reference standards for next-generation sequencing. Nat Rev Genet.

[14] Blackburn J, Wong T, Madala BS, Barker C, Hardwick SA, Reis ALM (2019). Use of synthetic DNA spike-in controls (sequins) for human genome sequencing. Nat Protoc.

[15] Knott GJ, Doudna JA (2018). CRISPR-Cas guides the future of genetic engineering. Science.

[16] Wu W, Yang Y, Lei H (2019). Progress in the application of CRISPR: from gene to base editing. Med Res Rev.

[17] COSMIC, the Catalogue of Somatic Mutations In Cancer [Internet]. Available from: https://cancer.sanger.ac.uk/cosmic. 10.1093/nar/gky1015 PMC6323903 30371878

[18] Kato M, Nakamura H, Nagai M, Kubo T, Elzawahry A, Totoki Y (2018). A computational tool to detect DNA alterations tailored to formalin-fixed paraffin-embedded samples in cancer clinical sequencing. Genome Med.

[19] NBDC Human Database [Internet]. Available from: https://humandbs.biosciencedbc.jp/en/.

[20] Okamoto S, Amaishi Y, Maki I, Enoki T, Mineno J (2019). Highly efficient genome editing for single-base substitutions using optimized ssODNs with Cas9-RNPs. Sci Rep.

[21] Zhu X, Xu Y, Yu S, Lu L, Ding M, Cheng J (2014). An efficient genotyping method for genome-modified animals and human cells generated with CRISPR/Cas9 system. Sci Rep.

[22] Sunami K, Ichikawa H, Kubo T, Kato M, Fujiwara Y, Shimomura A (2019). Feasibility and utility of a panel testing for 114 cancer-associated genes in a clinical setting: a hospital-based study. Cancer Sci.

[23] Williams HL, Walsh K, Diamond A, Oniscu A, Deans ZC (2018). Validation of the Oncomine() focus panel for next-generation sequencing of clinical tumour samples. Virchows Arch.

[24] Fisher KE, Zhang L, Wang J, Smith GH, Newman S, Schneider TM (2016). Clinical validation and implementation of a targeted next-generation sequencing assay to detect somatic variants in non-small cell lung, melanoma, and gastrointestinal malignancies. J Mol Diagn.

[25] Personalis. ACE CancerPlus Test for DNA & RNA Sequencing for Improved Patient Outcomes.https://www.personalis.com/labroots-ace-cancerplus-test-dna-rna-sequencing-improved-patient-outcomes/

[26] Lee A, Lee SH, Jung CK, Park G, Lee KY, Choi HJ (2018). Use of the ion AmpliSeq Cancer hotspot panel in clinical molecular pathology laboratories for analysis of solid tumours: with emphasis on validation with relevant single molecular pathology tests and the Oncomine focus assay. Pathol Res Pract.

[27] Paquet D, Kwart D, Chen A, Sproul A, Jacob S, Teo S (2016). Efficient introduction of specific homozygous and heterozygous mutations using CRISPR/Cas9. Nature.

[28] Yusa K, Rashid ST, Strick-Marchand H, Varela I, Liu PQ, Paschon DE (2011). Targeted gene correction of alpha1-antitrypsin deficiency in induced pluripotent stem cells. Nature.

[29] GGGnome. a fast and simple DNA sequence search engine GGGnome. p. http://gggenome.dbcls.jp/en/.

[30] Young M. A simple method to detect on-target editing or measure genome editing efficiency in CRISPR experiments. https://sg.idtdna.com/pages/education/decoded/article/a-simple-method-to-detect-on-target-editing-or-measure-genome-editing-efficiency-in-crispr-experiments.

[31] Lin YC, Boone M, Meuris L, Lemmens I, Van Roy N, Soete A (2014). Genome dynamics of the human embryonic kidney 293 lineage in response to cell biology manipulations. Nat Commun.

